# Later Chronotype Is Associated with Higher Alcohol Consumption and More Adverse Childhood Experiences in Young Healthy Women

**DOI:** 10.3390/clockssleep1010012

**Published:** 2019-02-12

**Authors:** Evelin Hug, Katja Winzeler, Monique C. Pfaltz, Christian Cajochen, Klaus Bader

**Affiliations:** 1 CBT Unit, Center for Specific Psychotherapy, Psychiatric Hospital of the University of Basel, Wilhelm Klein-Strasse 27, 4002 Basel, Switzerland; 2 Department of Consultation-Liaison Psychiatry and Psychosomatic Medicine, University Hospital Zurich, Haldenbachstrasse 18, 8091 Zürich, Switzerland; 3 University of Zurich, 8006 Zurich, Switzerland; 4 Centre for Chronobiology, Psychiatric Hospital of the University of Basel, Wilhelm Klein-Strasse 27, 4002 Basel, Switzerland; 5 Transfaculty Research Platform Molecular and Cognitive Neurosciences, University of Basel, 4055 Basel, Switzerland

**Keywords:** chronotype, social jetlag, adverse childhood experiences, alcohol, caffeine, women

## Abstract

This study aimed at examining potential associations of mid sleep timing (chronotype) and social jetlag with intake of alcohol and caffeine, depressive symptoms, and body mass index (BMI) in a sample of healthy young women. Furthermore, it was explored whether these behavioral sleep–wake parameters are associated with adverse childhood experiences (ACEs). In total, 146 women (21.7 ± 1.7 years) took part in a two-week assessment on daily consumption of alcohol and caffeine. They completed questionnaires on ACEs, chronotype, sleep quality and depressive symptoms. Partial correlations and Chi-Square tests were calculated to assess the relationships between the assessed variables. Results show an association on a trend level for chronotype (*r* = 0.162, *p* = 0.053) and a significant association for social jetlag (*r* = 0.169, *p* = 0.044) with average alcohol intake. Furthermore, participants with above-median ACEs were more likely to be late chronotypes compared to the below-median group (X2(2) = 6.595, *p* = 0.037). We could replicate the association among late chronotype, social jetlag and higher alcohol consumption in a sample of healthy, young women. Furthermore, our results suggest a relationship between ACEs and chronotype. Although it can be hypothesized that it is rather ACEs that have an impact on chronotype, further research is needed to explore this relationship more and to shed more light on the direction of the association between chronotype and ACEs as well as on underlying mechanisms and possible mediators.

## 1. Introduction

There are considerable inter-individual differences in self-selected sleep timing referred to as chronotype. The distribution of chronotypes within the population is almost normal and ranges from extremely early to extremely late chronotypes [1]. Early chronotypes wake up early in the morning and fall asleep early in the evening and vice versa late chronotypes fall asleep late in the evening and wake up late in the morning [2]. Chronotype is regulated by the sleep homeostat and the internal circadian clock, which promotes wakefulness during daytime and sleep at night and actively synchronizes with multiple and interacting Zeitgebers such as temperature, nutrition and light [3,4,5]. The latter has been shown to be the most important Zeitgeber for the entrainment of the endogenous circadian timing system [6,7]. Zeitgeber-strength, homeostatic build-up sleep pressure, age, sex and genetic variations in so-called “clock” genes are all factors, which lead to the above-mentioned inter-individual differences in chronotype [8,9,10,11,12].

In contrast to the normal distribution of chronotypes in the population, school- and work-times are fairly narrowly distributed with the major part of the working population of central Europe starting work between 07:00 and 09:00 [13]. People’s individual chronotypes can interfere with timing of working hours, school or social schedules. Late chronotypes might be forced to get up before their biologically driven wake-up time, whereas early chronotypes might have to stay up longer into their biological night. For late chronotypes, the combination of early wake-up times (controlled by external timing) along with late sleep onset (controlled by internal time) leads to the accumulation of sleep debt on workdays which will then be compensated for on work-free days by sleeping longer [14]. This discrepancy between internal and external timing is quantified by the absolute difference between midsleep on workdays and midsleep on work-free days and is referred to as social jetlag [15]. Although late chronotypes suffer more from social jetlag, early chronotypes do suffer from social jetlag as well, for example when early chronotypes stay up long into the night without the possibility of sleeping longer the following day due to their normal wake-up time [15].

Chronotype as well as social jetlag are associated with different adverse health consequences and unhealthy habits [16]. Late chronotypes report lower sleep quality and more daytime sleepiness [17,18,19], more depressive symptoms [20], and less healthy lifestyles and dietary habits [21,22]. Furthermore, they have a higher risk for type 2 diabetes [23], and consume more alcohol, nicotine, and caffeine [15,24]. Likewise, more social jetlag is associated with more depressive symptoms [20], increased body mass index (BMI) and obesity [25,26], as well as increased consumption of nicotine, caffeine, and alcohol [15].

Based on these findings, we aimed at examining potential associations between alcohol and caffeine intake, depressive symptoms and other health-related parameters (BMI and subjective sleep quality) and chronotype and social jetlag in a sample of healthy young women. Most research to date was conducted with male or mixed-gender study samples, mostly with a wide age range. It is well known that chronotype depends not only on genetics but also on age and sex [13]. In addition, findings by Fabbian et al. (2016) show that especially in women a tendency to later chronotype might act as an unfavorable aspect in the onset of mental or physical disorders [16]. Therefore, it is of great importance to examine possible associations in an all-female sample. Furthermore, we assessed daily alcohol and caffeine intake over 14 consecutive days instead of a single assessment of consumption as in most studies to date. As reported above, there is an association between nicotine consumption and chronotype as well as social jetlag. Data for this paper were taken from a larger study on acute stress, emotion regulation, and sleep in young female adults [27,28,29], from which heavy smokers had to be excluded because of interference with cortisol measures. However, there is a small number of light smoking participants (≤5 cigarettes/day; *N* = 23) in the sample and, therefore, smoking was included as a covariate in statistical analyses to account for possible confounding effects.

Additionally, we intended to explore whether social jetlag and chronotype are associated with adverse childhood experiences (ACEs) including general traumas as well as physical, emotional and sexual abuse. We and others have shown that exposure to early adversities is linked to changes in psychophysiological stress systems, such as changes in endocrine and cardiovascular stress responses [27,28,30,31]. Furthermore, ACEs are associated with adverse health outcomes such as neurological and musculoskeletal problems; respiratory, cardiovascular, and gastrointestinal disease; and unhealthy habits such as smoking and consuming alcohol [32,33]. A history of ACEs is linked to poor subjective sleep quality [34,35], frequent insufficient sleep [36], higher risk for sleep disturbances [37], and decreased sleep efficiency, prolonged sleep onset latency, and greater numbers of body movements during sleep [38,39]. However, to the best of our knowledge, it has not yet been investigated whether ACEs are linked to chronotype and social jetlag. Therefore, the second aim was to analyze whether there is a relationship between different parameters of sleep–wake behavior (chronotype and social jetlag), and ACEs in a sample of healthy young women.

## 2. Results

### 2.1. Sample Characteristics

Sample characteristics for age, ACEs, chronotype, social jetlag, actigraphy sleep measures, PSQI-score, alcohol intake, caffeine intake, symptoms of depression, and BMI, are depicted in Table 1 for the three chronotype groups (early, intermediate and late) separately. Both the average PSQI-score of 4.10 ± 1.8 (SD) and a mean sleep efficiency of 93.7% ± 5.1% (SD) indicated overall good sleep quality in our sample of healthy young women, which did not significantly differ between the three chronotype groups. Overall, there were 23 light smoking participants in the study. Out of those 23, two were in the group of early chronotypes, eight in the group of intermediate chronotypes, and 13 in the group of late chronotypes. A Chi-Square test of independence showed a significant association between chronotype groups and smoking (X2(2) = 8.931, *p* = 0.011).

### 2.2. Chronotype, Social Jetlag, and Intake of Alcohol and Caffeine

Pearson correlations and partial correlations with smoking as controlled covariate were calculated between mid-sleep on free days, sleep corrected (MSFsc) as well as social jetlag and alcohol and caffeine consumption, depressive symptoms, BMI and PSQI-score. A significant positive Pearson correlation was found for MSFsc and the average alcohol intake per day (*r* = 0.226, *p* = 0.006). When smoking was controlled for, a partial correlation on a trend level was found (*r* = 0.162, *p* = 0.053). No significant correlation was observed for chronotype and average caffeine intake per day (*r* = 0.136, *p* = 0.105).

There was a significant Pearson correlation between social jetlag and average alcohol intake per day (*r* = 0.216, *p* = 0.009). The correlation remained significant when smoking was controlled for (*r* = 0.169, *p* = 0.044). No significant correlation was observed for social jetlag and average caffeine intake per day (*r* = 0.030, *p* = 0.721).

Results did not change significantly when participants without any consumption of alcohol were excluded. No significant correlations were found between MSFsc or social jetlag and depressive symptoms, BMI, and PSQI-Score.

### 2.3. Chronotype, Social Jetlag, and Adverse Childhood Experiences

**Figure 1:**
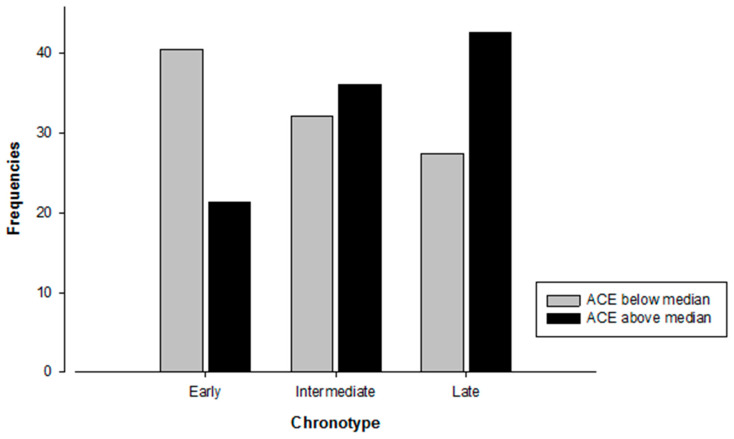
Number of participants in each distinct category of chronotype depending on ACE group affiliation. *N* = 145. Chi-Square test: X2(2) = 6.595, *p* = 0.037.

ACE groups were built by median split (*Mdn* = 3.00). A Chi-Square test of independence showed a significant association between chronotype groups and ACEs (X2(2) = 6.595, *p* = 0.037). Participants in the above median ACE group were less likely to be early chronotypes compared to participants in the below median ACE group (see Figure 1). 

In addition, results of a paired samples t-test indicate a significantly lower mean MSFsc in the below vs. the above median ACE group (*t*(143) = −1.995, *p* = 0.048). When using Pearson correlation, MSFsc correlated on a trend level with ACE total score (*r* = 0.145, *p* = 0.082). Nevertheless, partial correlation with smoking as covariate did not reach level of significance (*r* = 0.117, *p* = 0.162).

There was no significant association between social jetlag groups and ACEs (X2(1) = 1.558, *p* = 0.241). Means of social jetlag did not differ between the below and above median ACE group (*t*(143) = −0.472, *p* = 0.638). This result was confirmed when computing a Pearson correlation between social jetlag and ACE total score (*r* = −0.033, *p* = 0.692) as well as a partial correlation with smoking as covariate (*r* = −0.062, *p* = 0.462).

Furthermore, ACE groups did not differ in age, depressive symptoms, BMI, PSQI, alcohol intake, and caffeine intake. These results were confirmed when computing Pearson and partial correlations between ACE total score and the above-mentioned variables.

**Figure 2:**
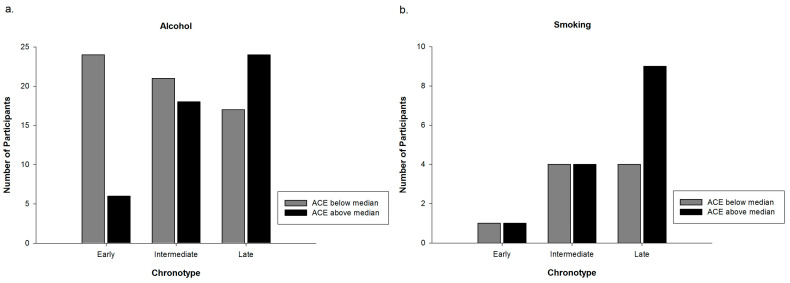
(**a**) Number of alcohol consumer in each distinct category of chronotype depending on ACE group affiliation. *N* = 110; and (**b**) number of smokers in each distinct category of chronotype depending on ACE group affiliation. *N* = 23.

Since smoking had an influence on the association between chronotype and alcohol consumption and ACEs, respectively, we calculated crosstabs for alcohol and nicotine consuming subgroups separately. Crosstabs for the relationship of ACE groups and Chronotype in the subgroups of alcohol as well as nicotine consumers were calculated for explorative reasons. Figure 2a,b shows the distribution of chronotype and ACE group in the consuming subgroups. 

## 3. Discussion

This study aimed to examine potential associations of chronotype and social jetlag with alcohol and caffeine intake, depressive symptoms and other health related parameters in a sample of healthy young women. The second aim was to explore whether there is a relationship between sleep–wake behavior as indexed by chronotype and social jetlag and ACEs.

In our sample of healthy young women, later chronotype and higher social jetlag were associated with more alcohol intake. These results corroborate and extend previous research [15,24], showing an association between chronotype as well as social jetlag and alcohol intake in an all-female study sample ranging in age from 18 to 25. One potential explanation for this relationship is the so-called “pub hypothesis”, stating that later chronotypes (who are also at a higher risk for suffering from social jetlag) simply have more time to drink alcohol in the evening due to a later bedtime and sleep onset [40]. The use of alcohol as a kind of self-medication to reduce physical tension (vegetative activation), which later chronotypes may experience to a higher degree than morning types in the evening hours when external social timing (e.g. working hours) requires relaxation to fall asleep, constitutes another explanation [24,40]. However, as our healthy women reported only low to moderate drinking (mean alcohol intake over all days in units M = 0.35, SD = 0.41), it can be hypothesized that rather the pub-hypothesis might explain the found association. Nevertheless, our findings are interesting and show that, even in a sample of healthy, young, women with low to moderate alcohol intake, effects between late chronotype, social jetlag and higher alcohol consumption were detectable. However, results also show that smoking, which was controlled for as covariate, has an effect on the relationship between chronotype, social jetlag and alcohol intake. This is in line with previous research that reported moderate correlations between smoking, chronotype and social jetlag, especially in the group of subjects of both gender aging 14–25 years [15]. Wittmann et al. (2006) reported that, in contrast to being a smoker or not, the number of cigarettes was not associated with chronotype or social jetlag. This corresponds with our finding of an association of even light smoking with later chronotype and social jetlag.

We did not find a significant association between chronotype or social jetlag and caffeine consumption. This is not in line with pervious findings showing a relationship between later chronotype as well as higher social jetlag and higher caffeine intake [24,40]. One reason for these discrepant findings may be methodological difficulties in measuring the units of substances. Especially in the case of caffeine, the units must be considered as rough estimates since caffeine concentration in beverages (especially tea and coffee) is dependent on their preparation and can differ substantially [15,41]. This methodological shortcoming decreases the comparability of different studies and might lead to different results [15,24]. Another reason for the absence of an association between chronotype, social jetlag and caffeine intake in the present study may be the young age of our participants (age range 18–25 years). It is known that caffeine consumption increases with age, reaching its peak around 50–64 years [41]. It is therefore possible that our participants consumed too little caffeine to detect potential associations.

Equally to the results for caffeine, associations between chronotype as well as social jetlag and BMI, depressive symptoms, and objective as well as subjective sleep quality did not reach significance. Other researchers have found associations between the above-mentioned variables in studies with mixed-gender samples with mostly wide age ranges [15,17,20,24,25,26]. The fact that the women in our study were all healthy, young and beyond that high functioning (no psychiatric disorders, mean years of schooling M = 12.5, 88% of all participants in tertiary education) may explain the absence of significant associations. This is clearly visible in the low scores of depressive symptoms, the average BMIs, and the very high subjective and objective sleep quality (see Table 1). However, it is also possible that multifactorial processes explain the link between late chronotype and higher social jetlag and different health variables. For example, Wittmann et al. presented in their work from 2010 a mediation model that supported the assumption that smoking and consumption of alcohol together mediate the relationship between later chronotype and depressive symptomatology [40]. Heavy Smoking (>5 cigarettes/day) was one of several exclusion criteria in our study, meaning that one important path of the model proposed by Wittmann et al. (2010) was not completely present but rather biased by the study design [40]. 

Results of the present study suggest a link between chronotype and ACEs. Participants having an ACE score above the median were more likely to be late chronotypes compared to participants having an ACE score below the median. Although a growing body of evidence shows a strong association of ACEs with different sleep characteristics [35,36,37,38,39], studies assessing the relationship between ACEs and chronotype are scarce. Barclay, Eley, Parsons, Willis, and Gregory (2013) showed in their cross-sectional study with adult monozygotic twin pairs that chronotype is largely genetically and biologically determined but that individual-specific non-shared environmental factors are related to differences in chronotype between twin pairs [42]. Negative life events in the past year, which were linked to a tendency towards eveningness, were one of these non-shared environmental factors. This association remained significant even in the monozygotic differences analysis, which indicates a purely non-shared environmental component of this association. Barclay et al. (2013) hypothesized that associations between non-shared environmental factors and chronotype are most likely bi-directional [42]. Regarding the association between chronotype and the experience of negative life events they suggest that this relationship might be mediated either by personality traits (neuroticism, risk-taking behavior, and externalizing behavior) or by poor sleep quality. Both are known to be associated with chronotype as well as with the experience of negative life events [35,38,39,43,44]. Our results provide further evidence for a relationship between environmental factors, namely ACEs, and chronotype. The additional benefit of our study is that we assessed negative life events in childhood and adolescence in a sample of healthy young women. This is a much wider lifespan than in Barclay et al. (2013) [42], and includes multiple sensitive periods of neurodevelopment, and might therefore be of particular interest in exploring the underlying processes of the association we found. It is known that the circadian and the stress systems are mutually interacting [45]. One hypothesis could therefore be that a history of ACEs may influence circadian regulation via interaction with the stress system (namely HPA-axis), especially when occurring during developmentally sensitive periods, and thus may contribute to variation in phenotype of chronotype. This might also explain the fact that ACEs are related to chronotype but not to social jetlag. From that perspective, ACEs can hardly be seen as a consequence of social jetlag, as this could be the case with alcohol consumption. However, clarifying this question thoroughly would need much more research. A second hypothesis could be the mediating role of sleep quality or personality traits in the relationship between chronotype and ACEs as proposed by Barclay et al. (2013) [42]. A closer look at the consuming subgroups of participants was taken post hoc, since results show that smoking had an influence on the relationship of chronotype with alcohol as well as ACEs. Figure 2a,b shows the distribution of alcohol consumers and smokers regarding chronotype and ACE group. Interestingly, the pattern of alcohol consumers seems to be the same as the one of the entire sample (cf. Figure 1), whereas the pattern of the small group of smokers appears to be different. Among smokers a difference between the ACE groups is only visible in late chronotypes. These results are not statistically confirmed and have only explorative character. Nevertheless, the pattern of smokers (Figure 2b) might indicate, that in participants of the above median ACE group, being a smoker might not only be explained by the pub hypothesis. It might also stand for a form of coping by having a short-term effect on emotion regulation and cognitive performance [40]. These different patterns of smokers and alcohol consumers are interesting but warrant further studies to explore possible associations and potential clinical implications.

### 3.1. Study Strengths

A strength of our study is the assessment of daily alcohol intake over 14 consecutive days using a palm handheld device instead of single assessment of consumption of stimulants in paper and pencil format. Computerized diaries are known to enhance compliance and to reduce recall bias in autobiographic memory retrieval [46]. This methodological advantage adds further strength to the suggested relationship of chronotype as well as social jetlag and alcohol intake. Furthermore, the confinement of the study sample to healthy young women free of psychiatric and somatic comorbidities allowed the investigation of associations between sleep–wake parameters and health related variables as well as ACEs in a homogenous, non-clinical sample.

### 3.2. Study Limitations

Due to our sample of healthy, young, non- or only light smoking, and high functioning women generalization is limited. Second, the range of ACEs and symptoms of depression may have been restricted due to the exclusion of participants with psychiatric diagnosis, potentially contributing to non-significant associations between variables. Third, subjective assessment of sleep–wake times has proven to be reliable in determining a person’s chronotype [47]. Nevertheless, there are potentially confounding variables such as age, sex, and season of assessment [12,47]. Age and sex were controlled for in the present study due to sample characteristics. Season of assessment is believed to influence phase of mid-sleep via photoperiod length (advancing mid-sleep when photoperiod gets longer) and was not controlled for in our sample [47]. To account for potential residual confounding it would be of great benefit to conduct further studies with larger samples. Finally, given the cross-sectional nature of the study it is not possible to draw any conclusion about cause and effect of the found relationships. 

The present study strengthens the assumption that late chronotype and higher social jetlag are associated with higher alcohol intake also in a sample of healthy, young women. There was also an influence of smoking in our sample with only very moderately smoking participants. Further analyses with ecological momentary assessment could be helpful to explore underlying processes and situational influences in the relationship between later chronotype as well as social jetlag and higher alcohol and nicotine consumption. It may also be helpful in studying consumption patterns and their relationship with chronotype and social jetlag. Additionally, there would be benefit in further exploring possible mediating effects of personality traits (e.g., novelty or sensation-seeking, impulsivity, and extraversion) since they are known to be linked to chronotype as well as consumption of psychoactive substances [24,48]. Furthermore, results indicate an association between chronotype and ACEs. Longitudinal research is needed to further explore this relationship and to shed more light on the direction of the association between chronotype and ACEs, on underlying mechanisms as well as on possible mediating effects of smoking. More knowledge about underlying processes might help to develop interventions preventing adverse health consequences resulting from late chronotype and ACEs by focusing on self-selected sleep timing, smoking and consumption of alcohol.

## 4. Materials and Methods 

### 4.1. Participants

Data were collected as part of a larger study on acute stress, emotion regulation, and sleep in young female adults [27,28,29]. The sample included 146 physically as well as mentally healthy young women (mean age 21.7 ± 1.7 years) who were recruited via flyers or emails at three schools for health care professions and social work in Basel, Switzerland. Participants were either working or going to school and followed five days of scheduled requirements, which started in the morning (approximately 07:00–09:00) and ended in the evening (approximately 16:00-17:00). Exclusion criteria for all participants included self-reported physical illness, psychiatric illness, pregnancy, regular and heavy tobacco use (>5 cigarettes a day), use of illicit drugs, use of any medication interfering with sleep, and working on night shifts. Participants received a monetary compensation of 150 CHF for their participation. The study was conducted in accordance with the Declaration of Helsinki and was approved by the local ethics committee. While all of the 146 participants completed the study, one individual was excluded for the present analyses because she did shift work in the month before our study and filled in the sleep questionnaire according to the shift timetable. Furthermore, the dataset of one participant was incomplete due to missing information in the electronic diary, reducing the sample size in the analyses concerning alcohol and caffeine consumption.

### 4.2. Procedure

Potential study participants contacted the study office by email or phone. All appointments took place in the laboratory of the cognitive behavioral therapy outpatient clinic of the Psychiatric Hospital of the University of Basel, Switzerland. During the first appointment, participants were screened on inclusion and exclusion criteria, provided written, informed consent, and filled in a standardized questionnaire about the experience of ACEs. Subsequently, they started two weeks of ambulatory assessment, which included questions about the daily consumption of alcohol and caffeine as well as actigraphy sleep measures (see Winzeler et al., 2014 for details about data obtained by actigraphy of this sample [29]). After one week of assessment, participants completed a standardized questionnaire to assess chronotype and social jetlag. After completion of the two weeks of ambulatory assessment, participants returned all material, filled in questionnaires about their subjective sleep quality as well as symptoms of depression, and were given their monetary compensation.

### 4.3. Measures

#### 4.3.1. Clinical Interview

A structured clinical interview for psychiatric disorders according to DSM-IV (SKID-I) was used to assess the absence of psychiatric illness [48]. All interviews were carried out by trained PhD students supervised by an experienced clinical psychologist.

#### 4.3.2. Chronotype and Social Jetlag

The Munich Chronotype Questionnaire (MCTQ) was used to assess timing of daily sleep [49]. To estimate chronotype, the midpoint between sleep onset and waking on free days (mid-sleep on free days, MSF) was calculated, and then corrected for potential excessive free-day sleep times due to sleep deprivation during work days (MSFsc; for correction algorithm, see supplement to the study by Roenneberg et al., 2004 [50]). Participants were grouped into distinct categories of chronotype according to their MSFsc score (early types: MSFsc ≤3.99; intermediate types: MSFsc 4.00–4.99; late types: MSFsc ≥4.99) [51]. The discrepancy between work and free days served as measure of “social jetlag”. It was computed by subtracting the MSW (midpoint of sleep on work days) from MSF.

#### 4.3.3. Alcohol and Caffeine

A menu-driven computerized questionnaire was developed to assess daily consumption of alcohol and caffeine. Palm Tungsten E handheld computers were used as recording devices. Questionnaires were programmed and displayed using Pendragon Forms 5.0 software (Pendragon Software Corporation, Buffalo Grove, IL, USA). 

Participants were instructed to report the number of units of alcohol as well as caffeine they consumed every day. For alcohol, one unit was defined as 300 mL of beer, one glass of wine or champagne, or one small glass of hard liquor. For caffeine, one unit was defined as one cup of coffee, one can of energy drink, two cups of tea or 600 mL of caffeinated soft drinks. Participants completed the question asking for caffeine and alcohol consumption on their handheld computers every evening bevor bedtime. 

Additional e-diary data included self-reported daily stress, pre-sleep arousal and subjective sleep measures and are reported elsewhere [29].

#### 4.3.4. Subjective Sleep Quality

The German version of the Pittsburgh Sleep Quality Index (PSQI) was used to assess subjective sleep quality and potential sleep problems [52,53]. Global scores of 5 or higher distinguish poor sleepers from good sleepers [52,53].

Sample characteristics of sleep efficiency and sleep onset latency are reported in Table 1 to give an impression on sleep quality in the present sample. For further information about actigraphy sleep measures and their methodical assessment in the present participants, see Winzeler et al. (2014) [29].

#### 4.3.5. Symptoms of Depression

Symptoms of depression were assessed with the German version of the Center for Epidemiological Studies Depression Scale (CES-D; German version: ADS-K) [54], which is well-established and has shown high internal consistency (Cronbach’s α = 0.90; 0.83 in the present sample) and test-retest reliability (*r* = 0.81).

#### 4.3.6. Adverse Childhood Experiences

Adverse childhood experiences (ACEs) before the age of 18 years were assessed with a German translation of the Early Trauma Inventory Self-Report questionnaire (ETI-SR) [55], which was designed to retrospectively assess adverse childhood experiences in four categories of childhood traumatic events. The questionnaire includes 31 items on general trauma (e.g., natural disasters and death of close person), 9 items on physical abuse, 7 items on emotional abuse, and 15 items on sexual abuse. Items for which a positive response is obtained are followed up with questions regarding frequency, age of onset, and impact at the time when the event happened. Events were summed up to a total score of occurred events (ACE total score), as Bremner et al., 2007 showed that alternate scores (weighting for severity, impact or age of onset) did not add to the validity of sum scores [55]. The ETI-SR has shown good internal consistency (Cronbach’s α = 0.78–0.90; 0.73 in the present sample) as well as validity in all trauma domains [55].

#### 4.3.7. Data Analysis

Analyses were performed using SPSS (version 23.0; SPSS, Chicago, IL). Prior to analysis, data were checked for outliers. To meet distributional assumptions, MCTQ, social jetlag, and ACE total score were transformed by natural logarithm. Mean alcohol consumption per day, mean caffeine consumption per day, and ADS-K scores were transformed by square root, and BMI was reciprocally transformed. 

Two-tailed Pearson correlations as well as partial correlations with smoking as controlled covariate were calculated to assess the relationships between chronotype, social jetlag, alcohol and caffeine consumption, depressive symptoms, BMI and PSQI-score. A Chi-Square test of independence was used to examine whether participants scoring above the ACE median were distributed differently across the three groups of chronotype (early, intermediate, late) compared to participants scoring below the ACE median. We also computed Pearson and partial correlations with smoking as covariate to cross-validate these results on a parametric level. For explorative reasons, crosstabs for the relationship of chronotype and ACE group in the subgroups of alcohol consumers as well as smokers were calculated and corresponding bar graphs are shown in the Results Section.

## 5. Conclusions

We have shown that late chronotype and higher social jetlag are associated with higher alcohol consumption in a sample of healthy, young women. In addition, we have found that there is an influence of smoking on this relationship even in our sample with only very moderately smoking participants. This points to the need of studying consumption patterns of both alcohol and nicotine together and their relationship with chronotype and social jetlag. Furthermore, our results suggest a link between ACEs and chronotype. Participants having a higher ACE score were more likely to be late chronotypes compared to participants having a lower ACE score. A history of ACEs may influence circadian regulation via interaction with the stress system (namely HPA-axis), especially when occurring during developmentally sensitive periods, and thus may contribute to variation in phenotype of chronotype. This might also explain the fact that ACEs are related to chronotype but not to social jetlag. From that perspective, ACEs can hardly be seen as a consequence of social jetlag, as this could be the case with alcohol consumption. However, this is quite speculative. Longitudinal research is needed to clarify this question more thoroughly and to shed more light on the direction of the association as well as on underlying processes and possible interventions preventing adverse health consequences resulting from late chronotype, ACEs, consumption of alcohol and smoking.

## Figures and Tables

**Table 1:** Sample Characteristics.

	Early Chronotype *N* = 47				Intermediate Chronotype *N* = 49				Late Chronotype *N* = 49			
	Mean	SD	Range		Mean	SD	Range		Mean	SD	Range	*p*
**Age (years)**	21.43	1.63	18–25		22.08	1.64	19–25		21.63	1.67	18–25	0.139
**ACE**	3.30	3.45	0–18		3.90	3.46	0–17		4.55	3.58	0–16	0.108
**MSFsc**	3.57	0.33	2.65–3.97		4.46	0.27	4.00–4.98		6.02	1.04	5.01–9.46	0.000 *
**Social Jetlag**	1.67	0.71	0.00–4.33		2.06	0.70	0.29–4.04		3.09	1.29	0.83–6.78	0.000 *
**Actigraphic sleep ^a^:**												
Sleep efficiency (%)	93.85 ^c^	2.29 ^c^	88.9–97.42 ^c^		93.47 ^d^	3.44 ^d^	78.73–97.43 ^d^		93.46 ^e^	2.81 ^e^	84.18–98.20 ^e^	0.735
Sleep latency (min.)	12.84 ^c^	4.91 ^c^	5.14–23.14 ^c^		13.23 ^d^	8.46 ^d^	3.29–51.75 ^d^		12.70 ^e^	5.96 ^e^	3.00–26.29 ^e^	0.930
No. of awakenings	3.96 ^c^	2.14 ^c^	0.71–9.29 ^c^		4.45 ^d^	2.69 ^d^	0.36–11.07 ^d^		4.39 ^e^	2.33 ^e^	0.71–11.50 ^e^	0.993
**PSQI-score**	3.91 ^f^	2.05 ^f^	0–8 ^f^		4.15 ^g^	1.77 ^g^	1–11 ^g^		4.33 ^h^	1.86 ^h^	0–8 ^h^	0.562
**Caffeine intake ^b^**	0.66 ^f^	0.70 ^f^	0–2.93 ^f^		0.91	0.99	0–4.64		1.00	0.90	0–4.57	0.074
**Alcohol intake ^b^**	0.24 ^f^	0.28 ^f^	0–0.93 ^f^		0.34	0.38	0–1.71		0.46	0.51	0–2.71	0.017 *
**ADS-K-Score**	7.26	5.27	0–25		6.90	5.31	0–21		6.16	4.66	0–21	0.674
**BMI**	22.38	4.07	18.7–38.8		21.75	2.83	17.6–33.8		22.31	3.31	18.4–34.4	0.644

*Note.* ACE, adverse childhood experience from Early Trauma Inventory Self-Report (ETI-SR); MSFsc, chronotype from Munich Chronotype Questionnaire MCTQ; PSQI, Pittsburgh Sleep Quality Index; ADS-K, German version of the Center for Epidemiological Studies Depression Scale; CES-D; ^a^ Mean values over all nights; ^b^ Average intake in units over 14 days; one unit of alcohol = 300 mL of beer/one glass of wine or champagne/one small glass of hard liquor; one unit of caffeine = one cup of coffee/one can of energy drink/two cups of tea/600 mL of caffeinated soft drinks ^c^ *N* = 44; ^d^ *N* = 45; ^e^ *N* = 43; ^f^ *N* = 46; ^g^ *N* = 47; ^h^ *N* = 48. * Significant differences (*p* < 0.050) between the three chronotype groups. *P*-values are presented as the result of one-way ANOVAs.

## Abbreviations

BMI	Body mass index
ACEs	Adverse childhood experiences
DSM-IV	Diagnostic and Statistical Manual of Mental Disorders, 4th edition
MCTQ	Munich Chronotype Questionnaire
MSF	Mid-sleep on free days
MSFsc	Mid-sleep on free days, sleep corrected
MSW	Midpoint of sleep on work days
PSQI	Pittsburgh Sleep Quality Index
CES-D	Center for Epidemiological Studies Depression Scale
ADS-K	German version of Center for Epidemiological Studies Depression Scale
ETI-SR	Early Trauma Inventory Self-Report Questionnaire
SD	Standard deviation
M	Mean
